# L-Threonine production from acetate by metabolically engineered Corynebacterium glutamicum using a pH-controlled fed-batch process

**DOI:** 10.1007/s00253-026-14083-7

**Published:** 2026-11-06

**Authors:** Philipp Broschard, Nina Endt, Astrid Wirtz, Christiane Glitz, Meike Baumgart, Michael Bott

**Affiliations:** 1 IBG-1: Biotechnology, Institute of Bio- and Geosciences, Forschungszentrum Jülich, 52428 Jülich, Germany; 2 The Bioeconomy Science Center (BioSC), Forschungszentrum Jülich, 52428 Jülich, Germany

**Keywords:** *Corynebacterium glutamicum*, L-Threonine, Acetate, Metabolic engineering, Fed-batch cultivation, Bioprocess

## Abstract

**Abstract:**

Acetate has a considerable potential as a non-food feedstock for microbial bioproduction, yet its use for amino acid production remains underexplored. Here, we developed a process for L-threonine production from acetate as the sole carbon source with *Corynebacterium glutamicum* by integrating rational, genome-based metabolic engineering with pH-coupled fed-batch cultivation. The engineered strain AT-3 carried five chromosomal mutations that alleviated feedback inhibition of key enzymes and redirected carbon flux toward the aspartate pathway. Substrate supply was controlled using a custom Visual Basic-based feeding script that automatically adjusted the acetic acid feed in response to online pH measurements. Robust process performance was maintained during a tenfold scale-up from 100-mL DASbox to 1-L DASGIP bioreactors. The optimized process achieved an L-threonine titer of 10.04 g/L and a yield of 0.086 g/g acetate. This study provides a basis for further studies on the development and improvement of acetate-based amino acid production process.

**Key points:**

• *Plasmid-free C. glutamicum strains produce L-threonine in acetate minimal medium*

• *pH-controlled acetic acid feed in bioreactors improved L-threonine production*

• *Acetate-based production of feed amino acids avoids competition with human food*

**Supplementary Information:**

The online version contains supplementary material available at https://doi.org/10.1007/s00253-026-14083-7.

## Introduction

Microbial production processes in industrial biotechnology are often designed to use starch hydrolysates from maize, wheat, or cassava or sucrose from sugar cane or sugar beets and therefore directly compete with the human food chain. To avoid this competition, a shift toward processes using non-food carbon sources, often obtained from industrial or agricultural waste streams, is a rapidly evolving field with industrial relevance (Wendisch et al. 2016). One such promising non-food carbon source is acetate, which can be utilized by many microbes. Although acetate is produced to about 90% by carbonylation of petrochemically derived methanol, the production of “green” acetate from renewable carbon sources like lignocellulosic biomass, organic waste, or gas fermentation is on the rise (Novak and Pflügl 2018). For example, the Austria-based company Lenzing AG produces bio-based acetic acid at ton scale as a by-product of beech wood processing in a sulfite pulp mill (De Wild 2015). Another path are gas fermentations in which acetogenic bacteria produce acetate from CO_2_ and H_2_ or syngas (Dürre and Eikmanns 2015; Takors et al. 2007).

In recent years, numerous studies reported on the use of acetate as carbon source for the production of value-added chemicals or proteins by various microorganisms (for reviews, see Kiefer et al. 2021b; Kim et al. 2021; Mutyala and Kim 2022). For example, a sequential fermentation was recently described in which first *Acetobacterium woodii* converted CO_2_ and H_2_ into acetate via the Wood–Ljungdahl pathway, after which the acetate-containing broth was used by dedicated *Corynebacterium glutamicum* strains for the production of L-glutamate, 3-hydroxypropionate, or mevalonate (Goldbeck et al. 2025). *C. glutamicum* serves as a workhorse in industrial biotechnology (Eggeling and Bott 2005), particularly as a cell factory for the large-scale production of amino acids, such as the flavor enhancer L-glutamate and the feed additive L-lysine (Eggeling and Bott 2015). These production processes use sugars as carbon source, but *C. glutamicum* was shown to utilize a broad range of alternative carbon sources, including acetate (Becker and Wittmann 2012; Blombach and Seibold 2010; Pérez-García et al. 2016; Wendisch et al. 2016). The utilization of acetate by *C. glutamicum* requires the glyoxylate cycle and has been extensively studied (Gerstmeir et al. 2003; Shah et al. 2018; Wendisch et al. 2000). Acetate enters the cell by passive diffusion (as acetic acid) and by active transport via the monocarboxylate transporter MctC, which is particularly important at alkaline pH (Jolkver et al. 2009). Besides the abovementioned example involving sequential fermentation, there are recent studies reporting on acetate-based production of itaconate (Merkel et al. 2022; Schmollack et al. 2022) and proteins (Kiefer et al. 2022) with *C. glutamicum*. With respect to amino acid production from acetate, L-glutamate production from acetate was reported (Goldbeck et al. 2025; Kanzaki et al. 1973). Most other studies used acetate as a co-substrate to sugars to produce L-lysine (Blombach et al. 2007; Paegle and Ruklisha 2003; Pelechová et al. 1980; Strehl et al. 2026) or L-valine (Bartek et al. 2011; Blombach et al. 2007). Overall, the potential of *C. glutamicum* for amino acid production from acetate as the sole carbon source remains largely unexplored.

In this study, we aimed to establish a proof-of-principle process for L-threonine production from acetate as sole carbon source. L-Threonine is an essential amino acid and produced industrially by fermentation with a market exceeding 700,000 metric tons per year (Wendisch 2020). L-Threonine is primarily used as a feed additive, as it is often a limiting amino acid in swine and poultry diets, while also finding applications in the food and pharmaceutical industries (Karau and Grayson 2014). *Escherichia coli *is currently the preferred host for L-threonine production, with reported titers up to 170.3 g/L in a semi-defined medium (Zhao et al. 2024b). With *C. glutamicum* strains, L-threonine titers of 57.7 g/L (Ishida et al. 1994) and recently of 78.3 g/L (Zhao et al. 2024a) were achieved using semi-defined media with glucose as major carbon source.

Despite the lower reported titers for L-threonine production from sugar-based carbon sources compared to *E. coli*, *C. glutamicum* bears process-related advantages. This species has the qualified presumption of safety (QPS) status of the European Food Safety Authority EFSA (EFSA BIOHAZ Panel, 2019). Feed-grade amino acids do not have to be chemically pure. In case of Biolys®, L-lysine is produced with *C. glutamicum* and after inactivation of the cells the entire fermentation broth is evaporated and thereby concentrated, followed by spray-drying and granulation (Eggeling and Bott 2015; Hermann 2003). L-Lysine (> 60%) is the principal active nutrient, while the remaining inactivated fermentation biomass provides additional nutritional value and is compatible with animal feeding. Furthermore, *C. glutamicum* tolerates high acetate concentrations of up to 60 g/L, although the growth rate gradually decreases above 10 g/L (Kiefer et al. 2021a), mainly due to the concentration- and pH-dependent uncoupling effect of acetate (Wolf et al. 2020). In this work, we created plasmid-free *C. glutamicum* ATCC 13032 strains to produce L-threonine from acetate using a chemically defined medium in a pH-based fed-batch process.

## Materials and methods

### Bacterial strains and cultivation media

Bacterial strains used in this study are listed in Table 1. *Escherichia coli* was routinely cultured in lysogeny broth (LB) or on LB agar plates at 37 °C, supplemented with 50 mg/L kanamycin if strains carried a plasmid. *Corynebacterium glutamicum* was cultured at 30 °C either in brain heart infusion (BHI, Difco, MD, USA) medium or on BHI agar plates, or in the original CGXII minimal medium (Keilhauer et al. 1993) but containing 0.03 g/L protocatechuic acid (CGXII_o_), or in a modified CGXII medium (CGXII_m_) in which the (NH_4_)_2_SO_4_ and 3-(N-morpholino)propanesulfonic acid (MOPS) concentrations were halved to 10 g/L and 21 g/L, respectively (Matamouros et al. 2023).

**Table 1:** Plasmids and strains used in this study

Strain or plasmid	Relevant characteristics	Source or reference
*E. coli* strains		
DH5α	F^−^Φ80*dlac*Δ(*lacZ*)M15 Δ(*lacZYA-argF*) U169 *endA1 recA1 hsdR17* (r_K_^−^ m_K_^+^) *deoR thi-1 phoA supE44 λ-gyrA96 relA1*; strain used for cloning procedures	(Hanahan 1983)
*C. glutamicum* strains		
WT	Wild type ATCC 13032; biotin-auxotrophic	DSMZ (Kinoshita et al. 1957)
DM1729	ATCC 13032 derivative with *lysC*^T311I^; served as template for the construction of pK19mobsacB-LysC^T311I^	(Georgi et al. 2005)
L1	WT derivative with *lysC*^T311I^ (cg0306)	This work
AT1	L1 derivative with *hom*^G378E^ (cg1337)	This work
AT2	AT1 derivative with exchange of the native *aspB* (cg0249) promoter by the *tuf* promoter of the elongation factor TU gene (cg0587)	This work
AT3	AT2 derivative with exchange of the native *lysC-asd* operon (cg0306-cg0307) promoter by the *tuf* promoter of the elongation factor TU gene (cg0587)	This work
Plasmids		
pK19mobsacB	Kan^R^, pK19 *oriV*_*Ec*_, *sacB*, lacZα, designed for allelic exchange in *C. glutamicum*	(Schäfer et al. 1994)
pK19mobsacB-LysC^T311I^	Kan^R^, plasmid for T311I mutation of LysC (cg0306)	This work
pK19mobsacB-Hom^G378E^	Kan^R^, plasmid for G378E mutation of homoserine kinase (cg1337)	This work
pK19mobsacB-P_*tuf*_::P_*aspB*_	Kan^R^, plasmid for exchange of the native *aspB* (cg0249) promoter by the promoter of the elongation factor TU gene (cg0587)	This work
pK19mobsacB-P_*tuf*_::P_*lysC*_	Kan^R^, plasmid for exchange of the native *lysC* (cg0306) promoter by the promoter of the elongation factor TU (cg0587) and for exchange of the native GTG start codon of *lysC* by ATG	This work

### Recombinant DNA work and construction of mutant strains

All plasmids and oligonucleotides used in this study are listed in Tables 1 and 2, respectively. DNA sequencing and oligonucleotide synthesis were conducted by Eurofins (Ebersberg, Germany). Standard methods such as DNA restriction and PCR were performed using established protocols (Green and Sambrook 2012). The host organism utilized for cloning was *E. coli* DH5α. Transformation of *E. coli* was performed using a heat-shock protocol (Hanahan 1983). Transformation of *C. glutamicum* was performed according to an electroporation protocol (van der Rest et al. 1999). Genomic mutations were integrated via a double homologous recombination using the pK19mobsacB plasmid (Schäfer et al. 1994). Chromosomal mutations were confirmed by colony-PCR using oligonucleotides binding up and downstream of the targeted genomic area. Correct integration was verified by sequencing of the PCR product.

**Table 2:** Oligonucleotides used in this study

Oligonucleotide name	Oligonucleotide sequence (5´→ 3´)
lysC_1	CGACGGCCAGTGAATTCGATTTCCGTGCCACGG
lysC_2	CAGGTCGACTCTAGAGGAGTTGCTGGTTTCCAGGG
Hom_F1_fw	GCCTGCAGGTCGACTCTAGAGGATCCCAGACGTCGAAGGC
Hom_F1_rv	GATCTTCCACATCCATGTCGAGGTGGTAC
Hom_F2_fw	GACATGGATGTGGAAGATCGCGTGGA
Hom_F2_rv	GTAAAACGACGGCCAGTGAATTCAATCCAGGAACTTCAGCGT
P_*tuf*_::P_*aspB*__F1_fw	GCCTGCAGGTCGACTCTAGAGGTACTTATCGATCATCCCGAC
P_*tuf*_::P_*aspB*__F1_rv	CATTCGCAGGGTAACGGCCAGCAGTGACCTCAAGCCAC
P_*tuf*_::P_*aspB*__F2_fw	GAAGTCCAGGAGGACATACAATGAGTTCAGTTTCGCTGCAG
P_*tuf*_::P_*aspB*__F2_rv	GTAAAACGACGGCCAGTGAATTCAGCAAATCCGCCCAAATC
P_*tuf*_::P_*lysC*__F1_fw	GCCTGCAGGTCGACTCTAGAGTCCAGTGCTGTCATGTCTCG
P_*tuf*_::P_*lysC*__F1_rv	CATTCGCAGGGTAACGGCCAGAGACAAAAGTGACAGGTGC
P_*tuf*_::P_*lysC*__F2_fw	GAAGTCCAGGAGGACATACAATGGCCCTGGTCGTACAGAA
P_*tuf*_::P_*lysC*__F2_rv	GTAAAACGACGGCCAGTGAATTACACCCTGGAAACCAGCAAC
P_*tuf*__F3_fw	TGGCCGTTACCCTGCGAATG
P_*tuf*__F3_rv	TGTATGTCCTCCTGGACTTC
P_*tuf*_::P_*aspB*__seq	CTTTAAACGCGCCAACCTGG
P_*tuf*_::P_*lysC*__seq	CGTCCGGTGCCTGCATAAAC
P_*tuf*__F3_seq	GCTCAGTCTTCCAGGCTGCTTATC

### BioLector cultivations

BioLector II or XT instruments (Beckman Coulter, Brea, USA) were used for small-scale cultivations of *C. glutamicum*. Cultivation was initiated by incubating a single colony in a seed culture (700 µL BHI in a 96-well plate, 900 rpm) for 6 to 8 h. Thereafter, the preculture was inoculated with 50 µL of the seed culture added to 750 µL CGXII_m_ medium supplemented with 40 g/L glucose. The main culture was inoculated to an optical density at 600 nm (OD_600_) of 1 using CGXII_m_ medium supplemented with 20 g/L sodium acetate. Cultivations were performed in 48-well FlowerPlates (Beckman Coulter, Brea, USA) at 30 °C, 1200 rpm and 85% humidity. Growth was measured online as scattered light at 620 nm (Kensy et al. 2009a, b) with a signal gain factor of 2. Growth rates were calculated over a minimum of 3 h in the exponential growth phase. After termination of the BioLector incubation, the cultures were centrifuged and the supernatant used for the determination of carbon sources and amino acids via HPLC analysis.

### Bioreactor cultivations

Bioreactor experiments were conducted in DASbox® or DASGIP® bioreactor systems (Eppendorf, Germany). The bioreactors were equipped with online probes for pH (EasyFerm Plus PHI K8 120/325, Hamilton, Switzerland) and dissolved oxygen (DO; VisiFerm DO ECS 120/325, Hamilton, Switzerland). DO was maintained above 30% by sequentially increasing the stirring rate of the two six-bladed Rushton turbine impellers (from 400 to 1000 rpm), the gas aeration rate (DASbox: from 4 to 10 sL/h; DASGIP: from 40 to 60 sL/h), and the oxygen fraction in the gas mixture. The temperature was set to 30 °C. The initial OD_600_ was adjusted to 1.0. The working volume was 0.1 L or 1 L for the DASbox and DASGIP bioreactors, respectively. Seed cultures were grown in 1 mL BHI medium for 6–8 h in a 96-deepwell plate at 900 rpm and 30 °C. The seed cultures were used to inoculate 50 mL CGXII_m_ precultures supplemented with 40 g/L glucose in 500-mL baffled shake flasks that were incubated for 18–20 h at 130 rpm and 30 °C. For bioreactor cultivations, CGXII medium without MOPS was used. Throughout the process, strong foaming was suppressed by the addition of antifoam 204 (Sigma-Aldrich). For batch bioreactor cultivations, the pH setpoint was 7 and controlled by the addition of 1 M H_2_SO_4_ or 1 M NaOH.

For fed-batch bioreactor cultivations, the medium was initially adjusted to pH 7.0 and no active pH control was applied during the first 0.5 h after inoculation. Subsequently, the pH was regulated by automated addition acetic acid (96%), which simultaneously served as the acetate feed. A custom feeding program was developed in Visual Basic and integrated into the DASware® control software. ChatGPT (OpenAI) was used to assist in the development and annotation of the DASware reactor scripting code. All final decisions and validation were made by the authors. The program has been adapted to different bioreactor systems and experiments by adjusting feed rates and control parameters. The adapted programs are provided in Table S1. This approach was inspired by the general principle described for a different MATLAB-controlled 42 L bioreactor system (Kiefer et al. 2021a).

### Quantification of amino acids and acetate by HPLC

For HPLC analyses, samples of culture supernatants were filtered through 0.2-µm filters (cytiva, Whatman, Puradisc 13) and, if necessary, diluted with deionized water. Amino acids were analyzed using an Agilent 1290 Infinity HPLC system (Agilent Technologies, Santa Clara, CA) equipped with a SecurityGuard™ ULTRA C18 precolumn (Phenomenex) and a Kinetex 1.7 µm EVO C18 100 Å separating column (100 × 2.1 mm; Phenomenex, Aschaffenburg, Germany). Samples were pre-column derivatized with *o*-phthaldialdehyde (OPA, Sigma-Aldrich, ready to use) by sequentially loading 2 µL OPA reagent, 1 µL sample, and 2 µL OPA reagent into the injection loop, followed by six mixing cycles and 1 min incubation, of which 1 µL was injected. The derivatized samples were separated at 40 °C The mobile phase consisted of buffer A (10 mM Na_2_HPO_4_, 10 mM Na_2_B_4_O_7_ · 10 H_2_O, pH 8.2) and methanol (buffer B) at a flow rate of 0.42 mL min⁻^1^ applying an increasing gradient of B (min/B%): 0.0 min/0% to 1.0 min/4.5% to 18.1 min/57%; 19.5 min/100%; 22.0 min/2%; 22 min end. Detection of derivatized amino acids was performed using a fluorescence detector (Agilent 1290 FLD; excitation/emission 340/450 nm), and quantification was based on external amino acid standards (Amino Acid Standard, AAS18; Sigma-Aldrich/Merck, Darmstadt, Germany), which were diluted in-house below 200 µM prior to analysis.

Acetate concentrations were determined using an Agilent 1260 LC system equipped with a Carbo-H Guard Cartridge and a Rezex™ ROA-Organic Acid H⁺ column (150 × 7.8 mm; Phenomenex, Aschaffenburg, Germany). Separation was performed at 60 °C for 25 min using 5 mM H_2_SO_4_ as the mobile phase at a flow rate of 0.5 mL min⁻^1^ and an injection volume of 5 µL. Detection was performed by refractive index detection (Agilent 1260 RID), and quantification was based on external standards measured before and after analytical samples.

## Results

### Construction of L-threonine overproducing strains

**Fig. 1:**
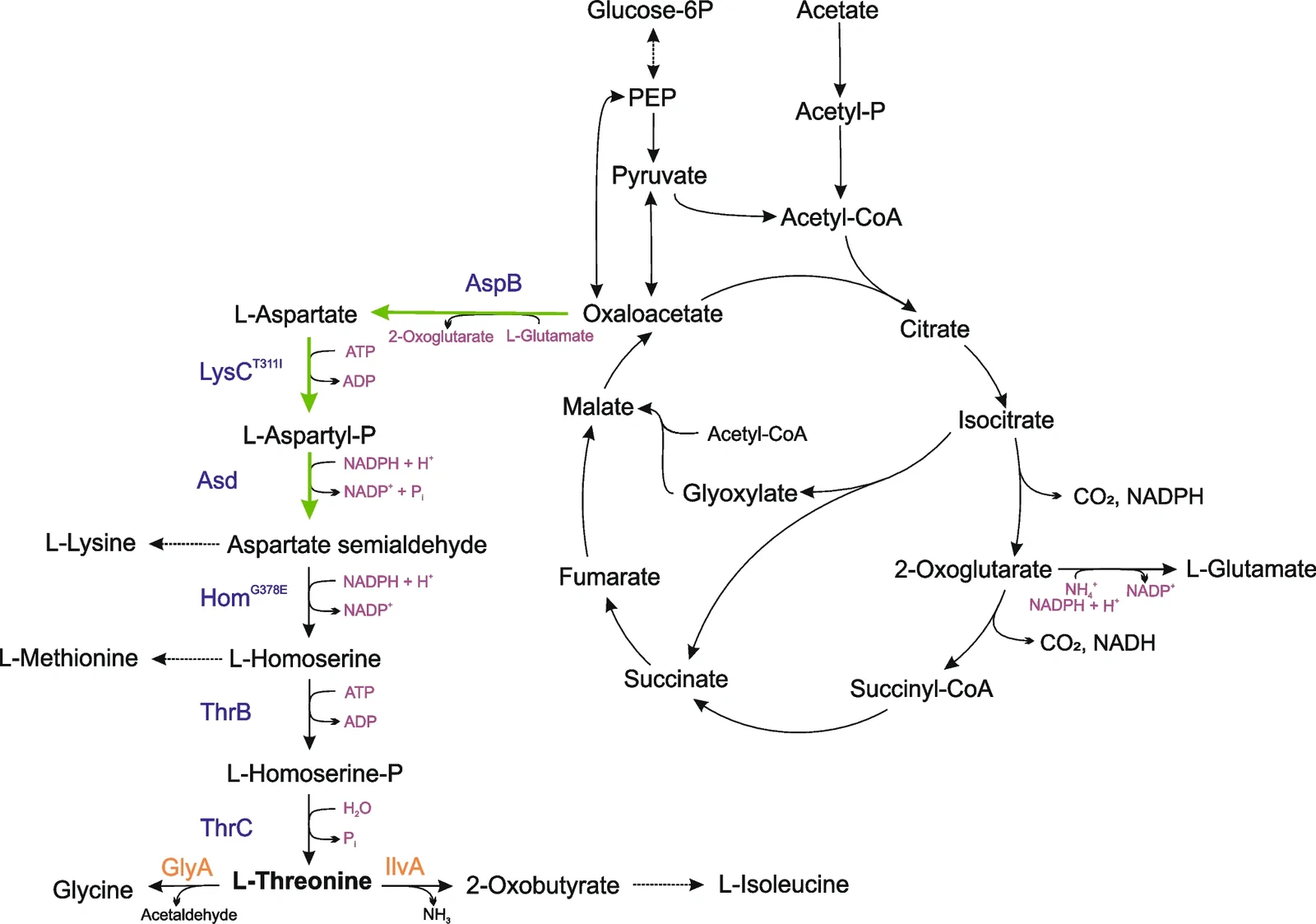
Scheme of metabolic pathways relevant for L-threonine production from acetate by *C. glutamicum*. Relevant enzymes for the conversion of oxaloacetate to L-threonine are depicted in blue: aspartate aminotransferase (AspB), feedback-resistant aspartate kinase (LysC^T311I^), aspartate semialdehyde dehydrogenase (Asd), feedback-resistant homoserine dehydrogenase (Hom^G378E^), homoserine kinase (ThrB), and threonine synthase (ThrC). Relevant cofactors or cosubstrates for these enzymes are depicted in magenta. Enhanced expression of genes in strains developed in this work is depicted by green arrows. Relevant competing pathways are depicted by dashed arrows, relevant competing enzymes in orange: serine hydroxymethyltransferase (GlyA) and threonine dehydratase (IlvA)

A *C. glutamicum* strain series starting from the type strain ATCC 13032 was constructed by introducing genomic mutations that had previously been shown to be effective for overproduction of the L-aspartate family of amino acids (AFAA), including L-lysine and L-threonine (Fig. 1). As a first step, the allosteric inhibition of aspartate kinase (encoded by *lysC*) by the combined action of L-threonine and L-lysine (Nakayama et al. 1966; Yoshida et al. 2007, 2010) was relieved by the amino acid exchange LysC^T311I^ (Ohnishi et al. 2002). Next the allosteric feedback inhibition of homoserine dehydrogenase (encoded by *hom*) by L-threonine (Archer et al. 1991; Nara et al. 1961) was abolished by the amino acid exchange Hom^G378E^ (Reinscheid et al. 1991). The resulting strain AT-1 subsequently served for bioprocess development.

During growth on acetate as sole carbon source, oxaloacetate is not only the precursor for L-threonine synthesis, but is also required to enable the TCA cycle, the glyoxylate cycle, and gluconeogenesis. To channel more oxaloacetate into the AFAA branch, the native promoter of aspartate aminotransferase (encoded by *aspB*) was exchanged with the strong constitutive *tuf* promoter (P_*aspB*_::P_*tuf*_) (Zhao et al. 2024b), resulting in strain AT-2. Furthermore, the native promoter of the *lysC-asd* operon was exchanged by the *tuf* promoter (P_*lysC*_::P_*tuf*_) and the *lysC* start codon was changed from GTG to ATG to improve translation efficiency (Becker et al. 2011), resulting in strain AT-3. Together, these modifications were expected to increase carbon flux into and through the aspartate pathway and L-threonine overproduction.

### Comparison of engineered strains with respect growth and L-threonine production in microscale cultivations

**Fig. 2:**
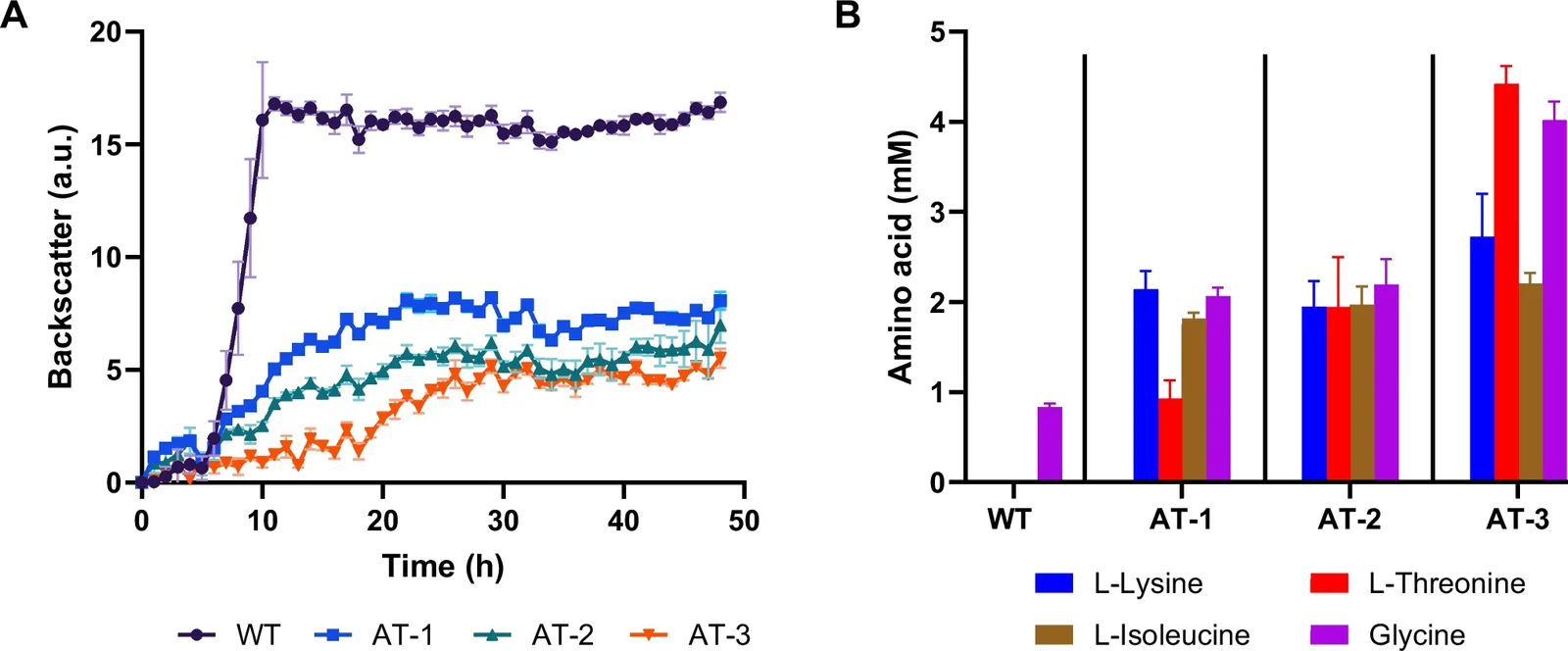
Growth (**A**) and amino acid production (**B**) of *C. glutamicum* WT and engineered strains in CGXII_m_ medium containing 20 g/L sodium acetate in a BioLector 2 system for 48 h at 30 °C, 1200 rpm, and 85% humidity. Amino acid concentrations were determined by HPLC. Mean values and standard deviations for three biological replicates are shown. a.u., arbitrary units

To assess the effects of the genetic alterations on growth and L-threonine production, the engineered strains and the wild type (WT) were cultured in a BioLector using CGXII_m_ medium supplemented with 20 g/L (244 mM) sodium acetate. The WT exhibited a growth rate of 0.57 h^−1^ and a final backscatter of 15.20 ± 0.53, whereas strains AT-1, AT-2, and AT-3 showed a strongly reduced growth rate and backscatter (Fig. 2, Table 3). The engineered strains were able to overproduce and secrete L-threonine. Strain AT-1 not only accumulated 0.93 ± 0.19 mM L-threonine, but also produced about 2 mM L-lysine, L-isoleucine, and glycine (Fig. 2). Strain AT-2 produced twice the amount of L-threonine (1.94 ± 0.55 mM), while L-lysine, L-isoleucine, and glycine remained comparable to strain AT-1. Strain AT-3 produced 4.42 ± 0.19 mM L-threonine besides 4.0 mM glycine, 2.7 mM L-lysine, and 2.2 mM L-isoleucine. This result confirmed that the metabolic engineering steps increased L-threonine production from acetate and shifted the product spectrum in favor of L-threonine.

**Table 3:** Growth characteristics of the indicated strains in the BioLector experiment in CGXII minimal medium with 20 g/L acetate as the sole carbon source

Strain	*µ* (h^−1^)	Final OD_600_
WT	0.57 ± 0.04	15.20 ± 0.53
AT-1	0.17 ± 0.01	6.17 ± 0.32
AT-2	0.15 ± 0.02	5.58 ± 0.92
AT-3	0.16 ± 0.01	4.12 ± 0.24

### Batch bioreactor cultivation of strain AT-1

**Fig. 3:**
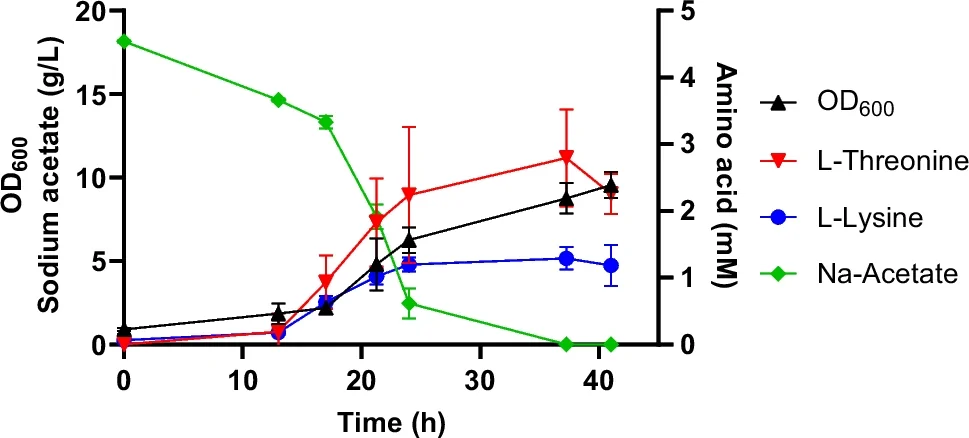
Batch cultivation of *C. glutamicum* strain AT-1 in a DASbox mini-bioreactor system. 100 mL CGXII_m_ medium was inoculated to an initial OD_600_ of 0.5. Cultures were grown at 30 °C with pH controlled at pH 7 and DO controlled to be above 30% by an intrinsic cascade. Amino acid and acetate concentrations were determined by HPLC. Mean values and standard deviations of two biological replicates are shown

To assess the transferability of BioLector results to stirred tank cultivation, batch experiments with strain AT-1 were conducted in an Eppendorf DASbox® mini parallel bioreactor system with a working volume of 100 mL using CGXII_m_ medium containing 20 g/L sodium acetate as sole carbon source (Fig. 3). The strain reached a final OD_600_​ of 9.55 ± 0.78 and a maximal L-threonine concentration of 2.79 ± 0.72 mM after 37 h, corresponding to a yield of 0.023 g/g acetate consumed. In contrast to the BioLector experiment, where L-lysine exceeded L-threonine overproduction for strain AT-1, the bioreactor cultivation resulted in a lower L-lysine titer (1.3 ± 0.2 mM). These results demonstrated that the controlled bioreactor cultivation promoted L-threonine overproduction and provided the basis for subsequent fed-batch process development.

### pH-based fed-batch bioreactor cultivation of strain AT-1

**Fig. 4:**
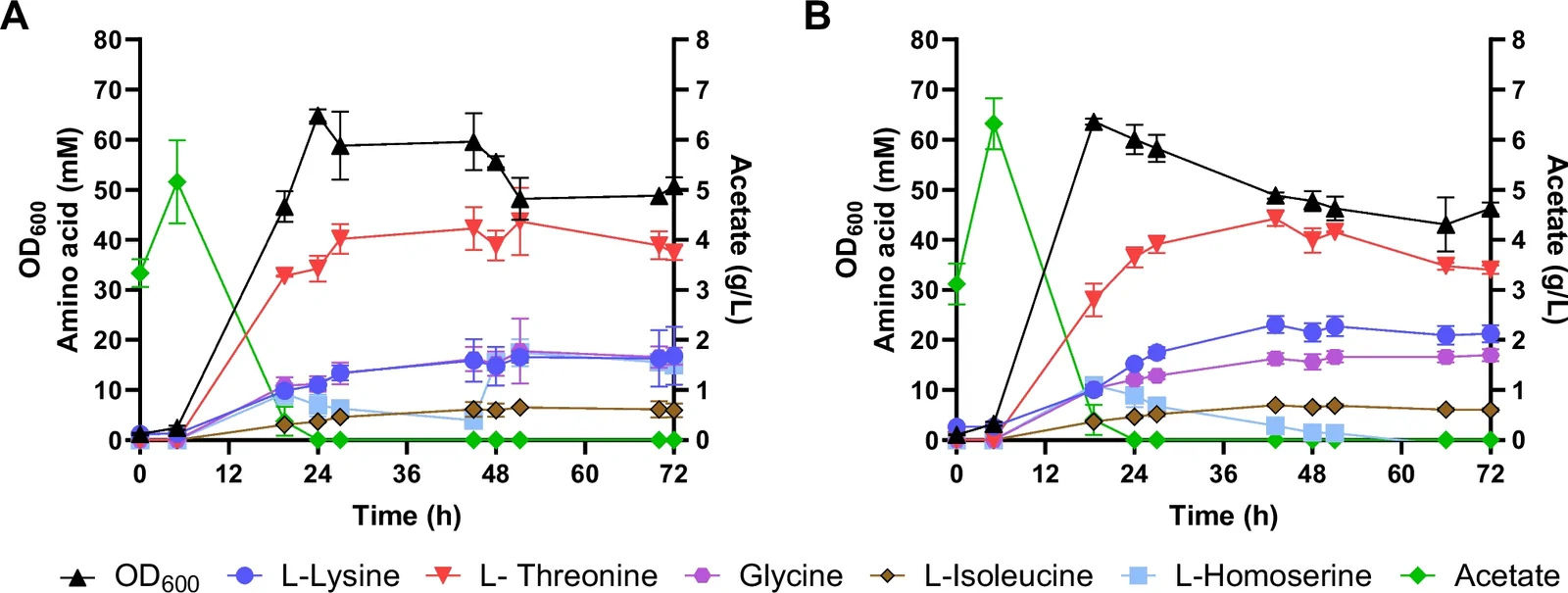
Fed-batch cultivation of *C. glutamicum* strain AT-1 in either DASbox (**A**) or DASGIP (**B**) bioreactor systems. Cultures were grown in 0.1 L (**A**) or 1 L (**B**) CGXII_m_ medium and inoculated to an OD_600_ of 1. Acetic acid feed was automatically added based on a custom-made Visual Basic script in a pH-based manner. Acetic acid (96%) was added when pH exceeded 7.15 until the pH dropped below this value. Cultivations were conducted at 30 °C and DO was maintained above 30% by an intrinsic cascade. Amino acid and acetate concentrations were determined by HPLC. Mean values and standard deviations for biological duplicates (**A**) and quadruplicates (**B**) are shown

To further increase L-threonine production, a pH-based fed-batch process was established for strain AT-1 in the Eppendorf DASbox mini bioreactor system. The process was designed to maintain low acetate concentrations throughout the cultivation to minimize its uncoupling effect. Acetic acid was automatically supplied to the bioreactor at a defined rate whenever the pH exceeded the specified threshold of pH 7.15, thereby serving both as carbon source and as pH-controlling agent (Fig. 4A). For this purpose, a custom-designed Visual Basic script compatible with the Eppendorf DASware control software was implemented. The fed-batch cultivation was conducted in CGXII_m_ with an initial sodium acetate concentration of 5 g L^−1^ (61 mM) and the pH-based feeding script was automatically started 0.5 h after inoculation. Strain AT-1 reached a maximum OD_600_ of 64.85 ± 1.20 after 24 h. Sodium acetate was not detectable after 24 h, indicating efficient substrate consumption and successful maintenance of low acetate concentrations. A maximal titer of 42.28 ± 4.31 mM L-threonine was obtained after 43 h. Compared to the batch process, the yield was more than doubled to 0.056 g/g acetate. As by-products, L-lysine, glycine, L-isoleucine, and L-homoserine were formed, but L-threonine was the predominant product throughout the cultivation. In comparison with the previous batch bioreactor process, fed-batch cultivation improved the process by maintaining low acetate concentrations and enabled elevated L-threonine formation and robust growth.

To evaluate the scalability of the process, the pH-based feeding script was then adapted to an Eppendorf DASGIP bioreactor system (Fig. 4B) with a working volume of 1 L, corresponding to a scale-up factor of 10 from the DASbox setup. Medium and process set points were held constant. The cultures reached a maximal OD_600_​ of 63.6 ± 0.56 after 18.5 h and the acetate profile was comparable to the one in the DASbox system. A maximal concentration of 44.26 ± 1.49 mM L-threonine was obtained after 43 h, corresponding to a yield of 0.054 g/g acetate. The by-product L-lysine accumulated to a slightly higher titer than in the DASbox system, whereas glycine and L-isoleucine reached comparable titers as in the DASbox system. Up to 10 mM L-homoserine was detected in the first 24 h, but then disappeared again. The observed growth and production profile indicated a successful transfer and scalability of the feeding concept.

### pH-based fed-batch bioreactor cultivation of strain AT-3

**Fig. 5:**
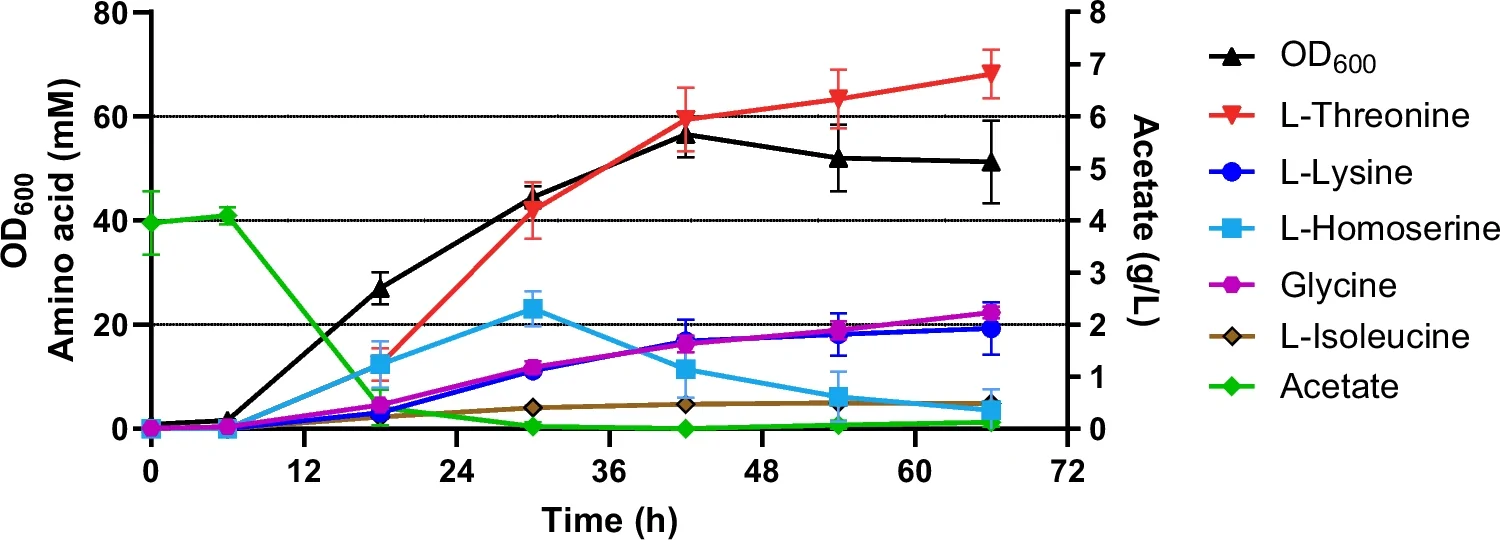
Fed-batch cultivation of *C. glutamicum* strain AT-3 in a DASGIP bioreactor system. Cultures were grown in 1 L CGXII_m_ medium inoculated to an OD_600_ of 1. Acetic acid (96%) feed was automatically added based on a custom-made Visual Basic script in a pH-based manner. Acetic acid was added when the pH exceeded 7.15 and stopped when the pH fell below this value. Amino acid and acetate concentrations were determined by HPLC. Cultivations were conducted at 30 °C and DO was maintained above 30%. Mean values and standard deviations of biological quadruplicates are shown

In the next step, we analyzed growth and amino acid production of strain AT-3 during fed-batch cultivation in the 1-L DASGIP bioreactor system (Fig. 5). Strain AT-3 grew slower than strain AT-1 and reached an OD_600_ of 56.50 ± 4.43 after 42 h, while acetate kinetics was comparable. L-Threonine production was particularly high between 24 and 48 h, reaching 60 ± 4.67 mM, followed by a slower increase to 68.12 ± 4.67 mM after 66 h. This corresponds to an improvement of 54% compared to strain AT-1. The yield also increased by 68.5% to 0.091 g/g acetate. In contrast to the increased L-threonine production, the formation of the by-products glycine and L-lysine was similar to that of strain AT-1. As observed for the parental strain, acetate was undetectable after 1 day of cultivation despite feeding throughout the cultivation, indicating that the process successfully maintained low acetate concentrations. Taken together, these results confirmed the transferability of the developed pH-controlled fed-batch process to higher engineered strains and confirmed the effectiveness of the metabolic engineering strategy.

### Optimization of the fed-batch cultivation of strain AT-3

**Fig. 6:**
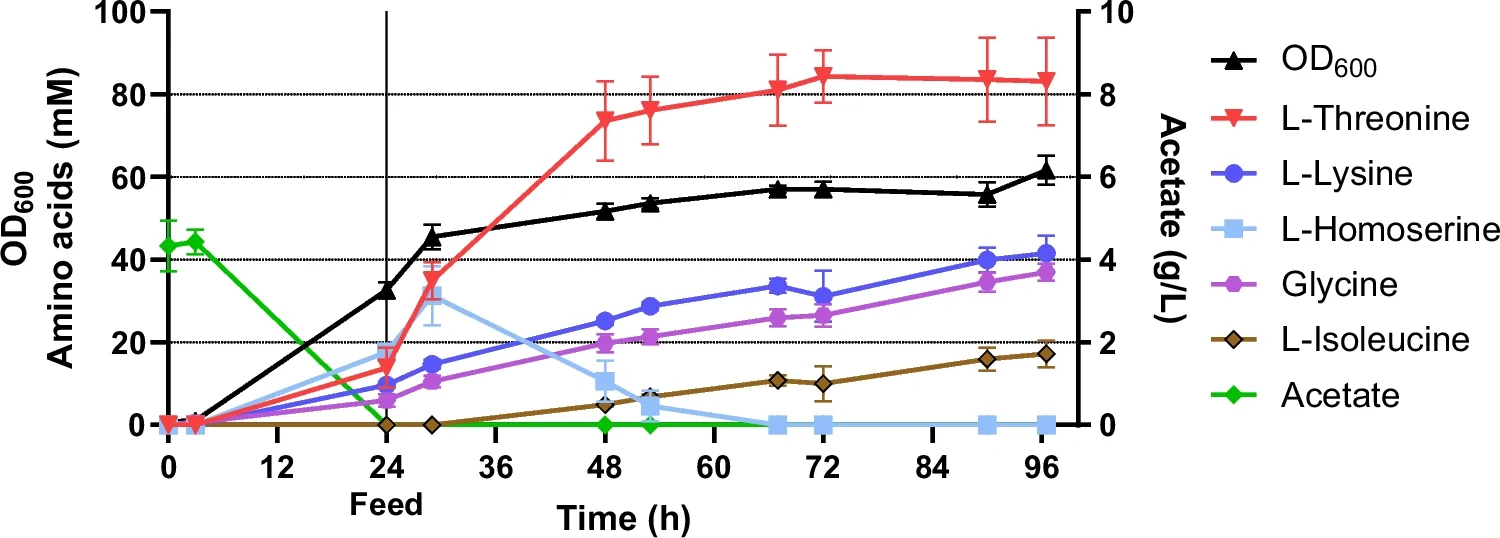
Fed-batch cultivation of *C. glutamicum* strain AT-3 in a DASGIP bioreactor system with constant sodium acetate feed. Cultures were grown in 1 L CGXII_o_ medium and inoculated to an OD_600_ 1. Acetic acid (96%) feed was added automatically based on a custom-made Visual Basic script when the pH exceeded 7.1 and stopped when the pH fell below this value. Continuous sodium acetate feeding (0.15 g h^−1^) was started after 24 h. Cultivation was performed at 30 °C and DO was maintained above 30%. Amino acid and acetate concentrations were determined by HPLC. Mean values and standard deviations of biological triplicates are shown

To further optimize the bioprocess, an additional continuous 0.15 g/L sodium acetate feed using a 300 g/L stock solution was implemented besides the acetic acid feed. In this way, acetate was provided also independent of the pH-controlled acetic acid feed and without medium acidification, thereby ensuring substrate availability. Based on previous acetate consumption profiles, the continuous sodium acetate feed was started after 24 h. In addition, we doubled the (NH_4_)_2_SO_4_ content of the medium by using CGXII_o_ instead of CGXII_m_ medium (Fig. 6). Compared to the previous process, L-threonine production followed a similar trend, but was further increased by 23.8% to a final titer of 84.30 ± 6.40 mM after 72 h (10.04 g/L), corresponding to a yield 0.086 g/g acetate. After 72 h, L-threonine production plateaued, while side products continued to increase, reaching approximately twice the concentration of the previous process by the end of the cultivation. OD_600_ increased until 29 h to 45.53 ± 2.99 and rose slower afterwards reaching 61.60 ± 3.46 by the end of the process. Despite the additional sodium acetate feed, no acetate accumulation was detected after 24 h and the anticipated low acetate concentration was maintained.

## Discussion

In this study, we demonstrated the feasibility of producing L-threonine from acetate as the sole carbon source using *C. glutamicum* through an integrated approach combining strain engineering and bioprocess development. We rationally engineered a series of plasmid-free L-threonine-producing strains through multistep genome-based metabolic engineering to relieve feedback inhibition of aspartate kinase and homoserine dehydrogenase and to increase oxaloacetate flux toward L-threonine. The engineered strains were characterized first in micro-cultivations and subsequently in pH-based fed-batch processes with DASbox and DASGIP bioreactor systems using acetic acid for pH control and substrate feed.

In BioLector cultivations, the metabolically engineered strains AT-1, AT-2, and AT-3 exhibited strongly reduced growth rates on acetate compared to the WT, but produced increasing concentrations of L-threonine of 0.93 mM (AT-1), 1.94 mM (AT-2), and 4.42 mM (AT-3). This result confirmed that the introduced genetic alterations (Table 1) were effective in triggering L-threonine overproduction. As further products, L-isoleucine, L-lysine, and glycine were formed. Biosynthesis of L-isoleucine starts from L-threonine as precursor (Fig. 1) and thus is an expected by-product. L-Lysine synthesis involves aspartate semialdehyde as precursor, similar to L-threonine, and homoserine dehydrogenase and dihydrodipicolinate synthase compete for this substrate (Fig. 1). In contrast to L-threonine, L-isoleucine, and L-lysine, glycine does not belong to the AFAA. Its strong synthesis is most likely caused by a side activity of the essential enzyme serine hydroxymethyltransferase (encoded by the *glyA* gene). Its main activity is the conversion of L-serine to glycine and methylene tetrahydrofolate; however, it also converts L-threonine to glycine and acetaldehyde. Purified serine hydroxymethyltransferase of *C. glutamicum* was shown to have an activity of 1.3 µmol min^−1^ (mg protein)^−1^ for conversion of L-threonine, which corresponds to 4% of the activity with L-serine as substrate (Simic et al. 2002). The acetaldehyde formed in this reaction can be oxidized to acetate by acetaldehyde dehydrogenase (Arndt et al. 2008).

Compared to microscale cultivation, strain AT-1 produced three times higher L-threonine levels when batch cultivated in DASbox bioreactors (Fig. 3). This increase might be attributed to the controlled pH and DO levels in the bioreactors. When acetate is used as sole substrate, ATP generation depends completely on electron transport phosphorylation and thus on a sufficient oxygen supply for respiration. The respiratory chain of *C. glutamicum* involves an energetically efficient cytochrome *bc*_1_-*aa*_3_ supercomplex branch and an energetically inefficient cytochrome *bd* oxidase branch, which is required under oxygen-limiting conditions (Bott and Niebisch 2003; Kabus et al. 2007). The importance of the cytochrome *bc*_1_-*aa*_3_ supercomplex for growth on acetate was demonstrated by the fact that a Δ*qcr* mutant lacking the *qcrCAB* genes for the cytochrome *bc*_1_ complex was hardly able to grow with acetate (Wolf et al. 2020). Furthermore, copper ions were reported to be required for maximal production of L-glutamate from acetate (Kanzaki et al. 1973), which can be explained by the fact that cytochrome *aa*_3_ oxidase (but not cytochrome *bd* oxidase) requires two copper ions for activity (Moe et al. 2022). Since microscale cultivation in BioLector instruments is known to cause oxygen limitation at higher cell densities, as reached with *C. glutamicum*, the better oxygen supply in the DASbox bioreactors is a likely explanation for the differences in the obtained L-threonine concentrations.

Building on the improved L-threonine production observed in controlled bioreactors, we next implemented a pH-based fed-batch strategy to further optimize the DASbox process with strain AT-1. Briefly, acetate consumption raised the culture pH, which automatically triggered a custom-built Visual Basic script to add acetic acid until the pH fell below the threshold set to pH 7.15. This pH-controlled feeding strategy maintained low acetate concentrations throughout the cultivation and resulted in a titer of 42.3 mM L-threonine after 43 h and a twofold increased yield (0.056 g/g) compared to the batch cultivation. This increase in yield might be attributed to the feeding regime which kept acetate concentration low, thereby minimizing the cellular stress that typically arises from acetate accumulation (Pinhal et al. 2019; Wolf et al. 2020). The DASbox fed-batch process with an initial culture volume of 100 mL was subsequently successfully scaled up by a factor of 10 to DASGIP bioreactors with an initial culture volume of 1 L, which led to comparable L-threonine titers (44.3 mM) and yields (0.054 g/g).

When strain AT-1 was replaced by strain AT-3 for the fed-batch process in the DASGIP bioreactors, an L-threonine titer of 68.1 mM and a yield of 0.091 g/g were achieved after 66 h, corresponding to increases of 54% and 69% compared to strain AT-1. Remarkably, the formation of the by-products glycine and L-lysine did not increase in the process with strain AT-3 compared to strain AT-1. A further improvement of L-threonine production was achieved by additional continuous feeding of sodium acetate (0.15 g/h) and increasing the nitrogen content of the medium. With this setup, an L-threonine titer of 84.3 mM (10.04 g/L) was reached after 72 h and a yield of 0.086 g/g. However, also the concentration of the by-products L-lysine, L-isoleucine, and glycine was almost doubled under these conditions by the end of the cultivation.

The concept of a pH-controlled acetic acid feeding strategy for fed-batch cultivation was previously successfully applied with a MATLAB script in a 42-L bioreactor for the production of biomass, GFP, or itaconate with *C. glutamicum* (Kiefer et al. 2021a, 2022; Merkel et al. 2022), confirming its functionality in pilot-scale production. The Visual Basic-based feeding script integrated with the Eppendorf DASware software established in our study is suitable for process development at smaller scales with simultaneous operation of multiple replicates within a single experiment, thereby increasing the experimental throughput.

In the past, numerous studies on the development of L-threonine production strains of *C. glutamicum* were reported (for reviews, see Dong et al. 2011; Sahm et al. 1996; Zhao et al. 2025) and identified many metabolic engineering targets. These studies typically used glucose with or without complex medium components as substrate for production. A recent publication (Zhao et al. 2024a) reported on the production of 78 g/L L-threonine from glucose with an engineered strain carrying an expression plasmid for the threonine exporter gene *thrE* (Simic et al. 2001) with a yield of 0.33 g/g glucose. Our study aimed to show the feasibility of L-threonine production from acetate as sole carbon source in minimal medium using *C. glutamicum* strains with maximally only five genomic modifications and carrying no plasmids. In fed-batch setups, we obtained up to 10 g/L L-threonine and a yield of 0.09 g/g within 72 h using only acetate for biomass and product generation. A recent study with a highly engineered *E. coli* strain carrying two plasmids reached a titer of 45.8 g/L L-threonine on acetate in 52 h with a yield of 0.22 mol/mol, which was said to be 65% of the maximal yield. A modified M9 medium with 2 g/L yeast extract and suitable antibiotics was used in a two-stage fed-batch fermentation in which the cells were first cultivated with a glucose batch before switching to acetate feeding (Vo et al. 2024).

In view of the fact that our *C. glutamicum* strain AT-3 contained only a limited set of engineered targets, we believe that further metabolic engineering of strain AT-3 offers a high potential to further improve the performance of the strain and the L-threonine yield, e.g., by increased expression of L-threonine biosynthesis and exporter genes and by reducing the formation of by-products. This includes e.g. the downregulation of *glyA* expression (encodes serine hydroxymethyl transferase), which has previously been shown to decrease glycine formation by 41% while increasing L-threonine accumulation by 49% (Simic et al. 2002). Another option is to introduce feedback-resistant variants of dihydrodipicolinate synthase (DapA), such as DapA^K68H^, that reduce L-lysine formation and can redirect carbon flux toward L-threonine biosynthesis without inducing L-lysine auxotrophy (Zhao et al. 2024a). Another metabolic engineering target is threonine dehydratase (IlvA), which catalyzes the first step in L-isoleucine biosynthesis by converting L-threonine to α-ketobutyrate. Reduction of the activity of threonine dehydratase could decrease L-isoleucine formation and enhance L-threonine accumulation without causing L-isoleucine auxotrophy (Möckel et al. 1994). Beyond strain engineering, further process optimization may represent an additional lever to enhance overall process performance, e.g., by using Raman spectroscopy (Grebe et al. 2026; Lee et al. 2004; Metcalfe et al. 2020) as an alternative or add-on to pH-based acetic acid feeding. In concert with the simplified downstream processing available for feed-grade amino acids produced with *C. glutamicum*, our study suggests that acetate-based production of L-threonine and other feed amino acids with *C. glutamicum* is an attractive option in the future.

## Supplementary Information

Below is the link to the electronic supplementary material.

ESM 1 (DOCX 22.8 KB)

## Data Availability

All data supporting the findings of this study are available within the paper and its Supplementary Information.
