# Supplementary material for: L-Threonine production from acetate by metabolically engineered Corynebacterium glutamicum using a pH-controlled fed-batch process
Source: Appl Microbiol Biotechnol. 2026 Nov 6;110:338. doi: 10.1007/s00253-026-14083-7 (PMC13739892; doi:10.1007/s00253-026-14083-7)
Supplement: Supplementary file 1 [file 253_2026_14083_MOESM1_ESM.docx]

**Supplementary Information**

**L-Threonine production from acetate by metabolically engineered *Corynebacterium glutamicum* using a pH-controlled fed-batch process**

Philipp Broschard^1^, Nina Endt^1^, Astrid Wirtz^1^, Christiane Glitz^1^, Meike Baumgart^1^, and Michael Bott^1,2^*

^1^ IBG-1: Biotechnology, Institute of Bio- and Geosciences, Forschungszentrum Jülich,

D-52428 Jülich, Germany

^2^ The Bioeconomy Science Center (BioSC), Forschungszentrum Jülich, D-52428 Jülich

*Corresponding author: [m.bott@fz-juelich.de](mailto:m.bott@fz-juelich.de)

**Table S1** Custom feeding program in Visual Basic integrated into the DASware® bioprocess control software (Eppendorf) for controlling pH-based acetic acid-feeding in bioreactor cultivations.

**DasBox AT-1**

Dim inhibitDuration_h As Double = 0.5

Dim pH_min As Double = 6.8

Dim pH_max As Double = 7.5

Dim feedRateLow As Double = 1

Dim feedRateMid As Double = 2

Dim feedRateHigh As Double = 10

Dim FeedRate As Double = 0

If P IsNot Nothing Then

With P

Select Case .Phase

Case 0

.Phase = 1

Case 1

If .PHPV >= pH_min And .PHPV <= pH_max Then

.PHActive = False

Else

.PHActive = True

End If

.PumpAActive = False

.PumpBActive = False

.PumpCActive = False

.PumpDActive = False

.FCSP = 0

.FDSP = 0

If .InoculationTime_H > inhibitDuration_h Then

.Phase = 2

End If

Case 2

If .PHPV >= pH_min And .PHPV <= pH_max Then

.PHActive = False

Else

.PHActive = True

End If

If .PHPV < pH_min Then

.PumpBActive = True

Else

.PumpBActive = False

End If

If .PHPV > 7.5 Then

FeedRate = feedRateHigh

ElseIf .PHPV > 7.3 Then

FeedRate = feedRateMid

ElseIf .PHPV > 7.15 Then

FeedRate = feedRateLow

Else

FeedRate = 0

End If

.FCSP = FeedRate

.PumpCActive = (FeedRate > 0)

If .PHPV > 7.5 Then

.PumpAActive = True

Else

.PumpAActive = False

End If

End Select

End With

End If

**DASGIP AT-1 / AT-3**

Dim inhibitDuration_h As Double = 0.5

Dim pH_min As Double = 6.8

Dim pH_max As Double = 7.5

Dim feedRateLow As Double = 5

Dim feedRateMid As Double = 10

Dim feedRateHigh As Double = 20

Dim FeedRate As Double = 0

If P IsNot Nothing Then

With P

Select Case .Phase

Case 0

.Phase = 1

Case 1

If .PHPV >= pH_min And .PHPV <= pH_max Then

.PHActive = False

Else

.PHActive = True

End If

.PumpAActive = False

.PumpBActive = False

.PumpCActive = False

.FCSP = 0

If .InoculationTime_H > inhibitDuration_h Then

.Phase = 2

End If

Case 2

If .PHPV >= pH_min And .PHPV <= pH_max Then

.PHActive = False

Else

.PHActive = True

End If

If .PHPV < pH_min Then

.PumpBActive = True

.PumpBActive = False

End If

If .PHPV > 7.5 Then

FeedRate = feedRateHigh

ElseIf .PHPV > 7.3 Then

FeedRate = feedRateMid

ElseIf .PHPV > 7.15 Then

FeedRate = feedRateLow

Else

FeedRate = 0

End If

.FCSP = FeedRate

.PumpCActive = (FeedRate > 0)

If .PHPV > 7.5 Then

.PumpAActive = True

Else

.PumpAActive = False

End If

End Select

End With

End If

**DASGIP AT-3 plus constant feed:**

Dim inhibitDuration_h As Double = 0.5

Dim startConstFeed_h As Double = 24

Dim constAcRate_ml_h As Double = 0.5

Dim pH_min As Double = 6.8

Dim pH_max As Double = 7.5

Dim feedRateLow As Double = 2

Dim feedRateMid1 As Double = 4

Dim feedRateMid2 As Double = 8

Dim feedRateHigh As Double = 20

Dim FeedRate As Double = 0

Dim BaseFeedRate As Double = 0

If P IsNot Nothing Then

With P

Select Case .Phase

Case 0

.Phase = 1

Case 1

If .PHPV >= pH_min And .PHPV <= pH_max Then

.PHActive = False

Else

.PHActive = True

End If

.PumpAActive = False

.PumpBActive = False

.PumpCActive = False

.PumpDActive = False

.FCSP = 0

If .InoculationTime_H > inhibitDuration_h Then

.Phase = 2

End If

Case 2

If .PHPV >= pH_min And .PHPV <= pH_max Then

.PHActive = False

Else

.PHActive = True

End If

If .PHPV < pH_min Then

.PumpBActive = True

Else

.PumpBActive = False

End If

If .PHPV > 7.5 Then

FeedRate = feedRateHigh

ElseIf .PHPV > 7.3 Then

FeedRate = feedRateMid2

ElseIf .PHPV > 7.2 Then

FeedRate = feedRateMid1

ElseIf .PHPV > 7.1 Then

FeedRate = feedRateLow

Else

FeedRate = 0

End If

.FCSP = FeedRate

.PumpCActive = (FeedRate > 0)

If .InoculationTime_H >= startConstFeed_h Then

BaseFeedRate = constAcRate_ml_h

Else

BaseFeedRate = 0

End If

.FDSP = constAcRate_ml_h

.PumpDActive = (BaseFeedRate > 0)

If .PHPV > 7.5 Then

.PumpAActive = True

Else

.PumpAActive = False

End If

End Select

End With

End If
